# Nano-Pulse Stimulation Therapy for the Treatment of Skin Lesions

**DOI:** 10.1089/bioe.2019.0027

**Published:** 2019-12-12

**Authors:** Richard Nuccitelli

**Affiliations:** Department of Biology, Pulse Biosciences, Hayward, California.

**Keywords:** nanosecond pulsed electric fields, nsPEF, NPS, Nano-Pulse Stimulation, skin, dermatology

## Abstract

Nano-Pulse Stimulation (NPS) therapy applies nanosecond pulsed electric fields to cells and tissues. It is a nonthermal modality that uses ultrashort pulses of electrical energy in the nanosecond domain. The cellular response to this therapy can be quite varied depending on the number of pulses applied and the total energy delivered. Reviewed in this study are some clinical trial data describing the effects of NPS therapy on normal skin as well as three different skin lesions as part of the first commercial application of this technology. NPS therapy has been found to clear seborrheic keratosis lesions with an 82% efficacy and sebaceous gland hyperplasia with a 99.5% efficacy. Pilot studies on warts indicated that 60% of the NPS-treated warts were completely cleared within 60 days. NPS therapy can be used to treat cellular lesions in the epidermis and dermis without affecting noncellular components such as collagen and fibrin.

## Introduction

Nano-Pulse Stimulation (NPS) therapy applies nanosecond-domain pulses of electrical energy to tissues. These pulses have been shown to specifically target cellular structures by driving water molecules into lipid bilayers of both the plasma membrane and internal organelles to form nanopores through which small molecules such as Na^+^, K^+^, or Ca^2+^ ions can flow. Since Ca^2+^ is maintained at very low levels inside cells compared with the extracellular concentration, extracellular Ca^2+^ will flow into the cell through nanopores to generate a transient increase or spike in the intracellular Ca^2+^ concentration.^1^ Ca^2+^ is a very important signaling molecule that is involved in the regulation of many different cellular functions, so it is not surprising that these pulses can have a variety of effects on cells from stimulating secretion in platelets^2^ to triggering regulated cell death in skin lesions and other cells.^3–5^ When a sufficient number of pulses is used, the regulated cell death cascade is triggered leading to reactive oxygen species generation,^6^ DNA fractionation,^7^ caspase 3 activation,^8,9^ and the translocation of calreticulin from the ER to the plasma membrane to serve as an “eat me” signal for dendritic cells.^9^ Many studies have shown that NPS therapy can trigger regulated cell death in both normal and malignant cells^10^ that can lead to the production of danger-associated molecular pattern molecules that in turn can initiate an immune response.^8,9,11^ The first commercial application of this technology will be for the elimination of unwanted skin lesions, which will be described in this study.

## Results

### Characteristics of NPS

The energy delivered by a pulsed electric field is determined by the product of the applied voltage, current, and pulse width. Owing to the short pulse width, pulses in the nanosecond domain deliver much less energy than more conventional micro- or millisecond-long pulses. That means that they are generally nonthermal so that their effect on cells is quite different from that of the longer conventional pulses. Their short duration allows them to penetrate into the cell interior before cellular ions can respond to the imposed field with a rearrangement that generates an equal and opposite internal field resulting in zero net electric field within the cell. Once inside the cell, these pulses can act directly on organelles to generate nanopores in their surrounding membranes as well. Of course, that will only occur if the field across the organelles is sufficient to force water defects into those lipid membranes. That is why NPS technology uses fields on the order of 30 kV/cm, which can generate the voltage drop of 1 V required to form nanopores across a submicron diameter organelle.

### Effects on normal skin

**FIG. 1.**
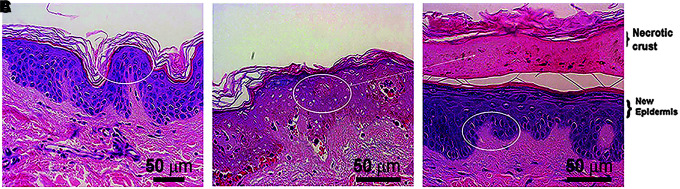
H&E-stained sections of abdominal skin fixed at different times. **(A)** Skin fixed before NPS treatment; **(B)** 1 day after treatment; **(C)** 1 week after treatment. The oval ring marks the healthy epidermis in **(A)** and **(C)** as well as the dying epidermis in **(B)** with nuclei that do not stain with H&E. This layer in **(B)** comes off as a necrotic crust one week later in **(C)**. Reprinted with permission from Kaufman et al.^12^ H&E, hematoxylin and eosin; NPS, Nano-Pulse Stimulation.

When NPS therapy is applied to normal abdominal and facial skin that is scheduled for resection, histological analysis can be performed after resection to determine the effects of the NPS treatment on skin structures^12^ (Fig. 1). Within 1 day, the epidermis exhibits signs of regulated cell death that include the lack of nuclear staining with standard hematoxylin and eosin exposure to thin sections of tissue. One week later, this dead epidermal layer peels off the skin as a crust or scab revealing a regenerated epidermis below. By using the appropriate NPS therapy, the epidermis is regenerated without scarring and any lesion residing in the epidermis can be readily removed.

### Effects on epidermal lesions

**FIG. 2.**
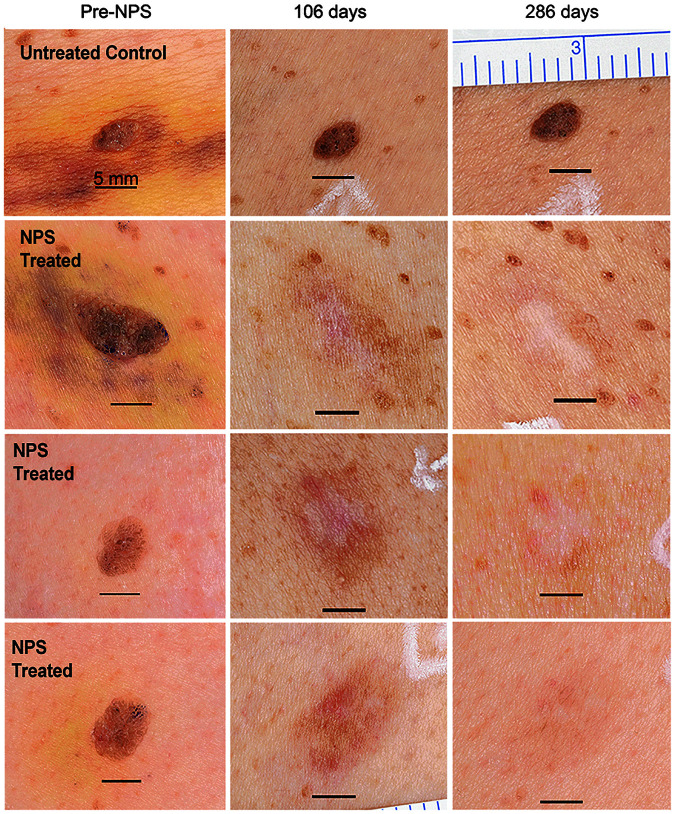
Pigmented seborrheic keratoses from a single subject shown before (Pre-NPS) a single NPS treatment of 2.5–10J as well as two timepoints after treatment. Black scale bar indicates 5 mm. Reprinted with permission from Hruza et al.^13^

Seborrheic keratosis (SK) is an example of a lesion residing in the epidermis and the results of a clinical trial (approved by Biomedical Research Institute of America) treating SK with NPS therapy have recently been published.^13^ Using treatment times of less than a minute, a process of regulated cell death is initiated and the lesion typically is cleared within a few weeks. One figure from that publication is included in this study (Fig. 2). Immediately after NPS treatment, edema and erythema are evident, and a crust is formed over the following week. The crust usually peels off within a week taking the lesion with it and hyperpigmentation is often present but fades over the following months. NPS therapy cleared >82% of the 174 lesions with a single treatment. These data also showed that NPS therapy had no effect on fibrous components of the skin such as collagen, fibrin, and elastin.

### Effects on dermal lesions

**FIG. 3.**
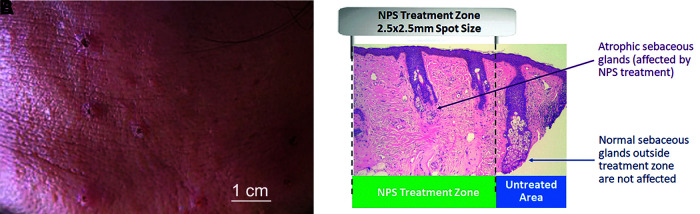
**(A)** Several SGHs on the forehead; **(B)** H&E-stained section of SGH taken 30 days post-treatment showing regulated cell death of the sebaceous glands located in the treatment zone. One sebaceous gland located outside of the treatment region is unaffected. SGH, sebaceous gland hyperplasia.

**FIG. 4.**
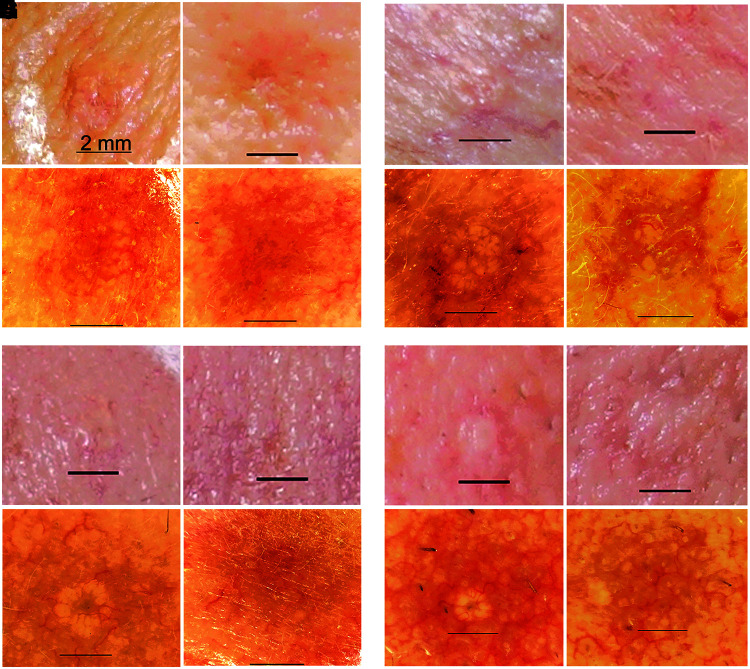
Pairs of images from four SGHs taken before **(A, C, E, G)** and 60 days after NPS treatment **(B, D, F, H)** from four different patients. Upper photo in each pair is the reflected light image showing skin surface and lower photo is the dermatoscope image of the same region showing sebaceous glands. Scale bar in each image is 2 mm long. Reprinted with permission from Munavalli et al.^14^

Sebaceous glands reside in the dermis and at times can proliferate to form a sebaceous gland hyperplasia (SGH) that appears as a raised papule on the skin, often with a depression in the center (Fig. 3A). NPS treatment of facial skin has been found to stimulate regulated cell death in sebaceous glands (Fig. 3B). A nonsignificant risk clinical trial (approved by Biomedical Research Institute of America IRB) treating 222 of these lesions with 0.9–0.5 J resulted in clearance in 99% of the lesions within 60 days^14^ (Fig. 4). Only 18 of these lesions required a second treatment and that was usually due to missed targeting on the first treatment. Thirty-two percent of these treated lesions were noted as having a slight volume loss in this first trial, but ongoing studies show a reduction in this volume loss when lower NPS energies are applied.

### Warts

One of the most challenging skin lesions to treat is the common wart due to its high rate of reoccurrence after treatment. Warts are usually caused by human papilloma virus 1 or 2 and are commonly treated by freezing with liquid nitrogen. This treatment usually results in partial shrinkage of the wart, but it often grows back.

**FIG. 5.**
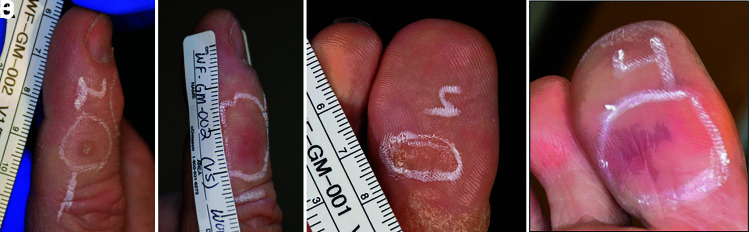
**(A)** Finger wart before NPS treatment; **(B)** 60 days after two treatments of 7.5 J; **(C)** Toe wart before NPS treatment; **(D)** 120 days after one NPS treatment of 7.4 J.

**FIG. 6.**
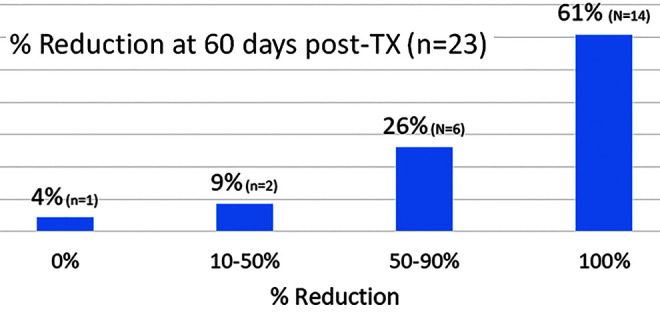
Distribution of the size reduction for 23 warts measured 60 days after NPS treatment.

Pilot trials treating warts on the hand and foot using NPS therapy have been providing promising results (Figs. 5, 6). Most of the warts treated were recalcitrant and failed to be cleared after one or more liquid nitrogen treatments. However, a majority of these recalcitrant 23 warts treated with NPS therapy were 100% cleared at 60 days post-treatment (Fig. 6). A pivotal trial with many more subjects is underway.

## Discussion

The main advantages of NPS technology is its specificity for cells and its nonthermal mechanism of action. That enables the clearance of skin lesions without perturbing the dermal collagen that could lead to scarring. Thus, the cellular epidermis as well as cellular components of the dermis are specifically targeted by NPS therapy. The epidermis typically forms a crust that comes off along with any lesions within it, uncovering a regenerated epidermis. Similarly, cellular structures in the dermis can be affected by NPS therapy, providing a good approach to eliminate SGH and possibly acne.

The mechanism used by NPS technology to initiate regulated cell death is unique. By stimulating the pathway of regulated cell death or apoptosis that is normally used by all cells at the end of their useful life, NPS technology gives the instruction to initiate this pathway and the cells do the rest. That leads to the clean removal of the treated lesions since they undergo their natural programmed cell death process with one step in that process being the expression of calreticulin on the cell surface as an “eat me” signal to dendritic cells. Those cells remove the dying lesion cells cleanly without scarring.

## Conclusion

NPS technology has been shown to be very effective in the clearance of many types of cellular skin lesions while sparing the noncellular components of the dermis. The proper energies can provide scarless lesion elimination with short treatment times and very high efficacy. This is a physical modality that will have the same effect of generating nanopores in both the organelles and plasma membrane of all cell types. The cellular response depends on the amount of energy delivered and can be quite diverse. This first application of NPS technology to dermatology utilizes its ability to trigger regulated cell death in cellular targets resulting in the scarless clearance of unwanted skin lesions in both the dermis and epidermis.
